# Evaluating the effect of inequalities in oral anti-coagulant prescribing on outcomes in people with atrial fibrillation

**DOI:** 10.1093/ehjopen/oeae016

**Published:** 2024-03-05

**Authors:** Ryan J Mulholland, Francesco Manca, Giorgio Ciminata, Terry J Quinn, Robert Trotter, Kevin G Pollock, Steven Lister, Claudia Geue

**Affiliations:** University of Glasgow, Glasgow, UK; University of Glasgow, Glasgow, UK; University of Glasgow, Glasgow, UK; University of Glasgow, Glasgow, UK; Pfizer UK, Surrey, UK; Bristol Myers Squibb, Uxbridge, UK; Bristol Myers Squibb, Uxbridge, UK; University of Glasgow, Glasgow, UK

**Keywords:** Atrial fibrillation, Stroke, Pharmacoepidemiology, Real-world data

## Abstract

**Aims:**

Whilst anti-coagulation is typically recommended for thromboprophylaxis in atrial fibrillation (AF), it is often never prescribed or prematurely discontinued. The aim of this study was to evaluate the effect of inequalities in anti-coagulant prescribing by assessing stroke/systemic embolism (SSE) and bleeding risk in people with AF who continue anti-coagulation compared with those who stop transiently, permanently, or never start.

**Methods and results:**

This retrospective cohort study utilized linked Scottish healthcare data to identify adults diagnosed with AF between January 2010 and April 2016, with a CHA_2_DS_2_-VASC score of ≥2. They were sub-categorized based on anti-coagulant exposure: never started, continuous, discontinuous, and cessation. Inverse probability of treatment weighting-adjusted Cox regression and competing risk regression was utilized to compare SSE and bleeding risks between cohorts during 5-year follow-up. Of an overall cohort of 47 427 people, 26 277 (55.41%) were never anti-coagulated, 7934 (16.72%) received continuous anti-coagulation, 9107 (19.2%) temporarily discontinued, and 4109 (8.66%) permanently discontinued. Lower socio-economic status, elevated frailty score, and age ≥ 75 were associated with a reduced likelihood of initiation and continuation of anti-coagulation. Stroke/systemic embolism risk was significantly greater in those with discontinuous anti-coagulation, compared with continuous [subhazard ratio (SHR): 2.65; 2.39–2.94]. In the context of a major bleeding event, there was no significant difference in bleeding risk between the cessation and continuous cohorts (SHR 0.94; 0.42–2.14).

**Conclusion:**

Our data suggest significant inequalities in anti-coagulation prescribing, with substantial opportunity to improve initiation and continuation. Decision-making should be patient-centred and must recognize that discontinuation or cessation is associated with considerable thromboembolic risk not offset by mitigated bleeding risk.

## Graphical Abstract

**Graphical Abstract:**
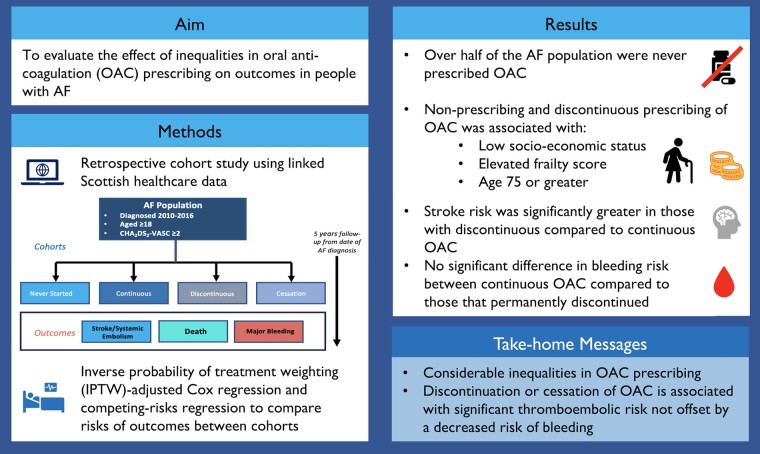

## Introduction

### Background/rationale

Despite the high thromboembolic risk in the atrial fibrillation (AF) population, stroke prophylaxis with anti-coagulants is frequently under-utilized or prematurely discontinued, generally due to the monitoring requirements of warfarin or a perceived high risk of bleeding.^1^ Indeed, the likelihood of older adults and females being anti-coagulated is paradoxically lower, despite their higher stroke risk.^2^ A prior observational study of people with non-valvular AF (NVAF) prescribed warfarin in a national data set combining Medicare and insurance claims data reported that the risk of ischaemic stroke is approximately doubled in those that discontinued warfarin compared with those with continuous prescriptions.^3^ A study of 1361 individuals with NVAF prescribed an alternative vitamin K antagonist, acenocoumarol, at an anti-coagulation centre in Spain suggested that cessation of anti-coagulation is associated with increased stroke, adverse cardiovascular events, and all-cause mortality.^4^ Whilst the acute treatment of those with bleeding associated with anti-coagulation often requires immediate discontinuation of anti-coagulation, the ongoing management is complex; clinical decision-making must consider the competing risks of a thromboembolic event if anti-coagulation is withheld and a recurrent bleed if it is recommenced, and clinical consensus is currently lacking as to the optimal approach.^5^ Whilst there is an increasing body of literature around patterns of adherence to anti-coagulation, few studies have evaluated the clinical outcomes associated with discontinuation of anti-coagulation in individuals with AF, particularly in the context of a major bleeding event.

All individuals in Scotland are assigned a unique identification number, the Community Health Index (CHI) number, creating a record of engagements with health and social care facilities through the lifetime.^6,7^ Linkage of national databases by CHI number affords the opportunity to analyse rich Scotland-wide individual patient data; this real-world data may be leveraged to explore research questions, such as the impact on clinical outcomes of discontinuing oral anti-coagulation (OAC) in people with AF for which a randomized controlled trial may be infeasible.^8–11^

### Objectives

The primary objective of this study was to evaluate the effect of inequalities in anti-coagulant prescribing by comparing the risks of stroke/systemic embolism (SSE) in adults with AF with discontinuous exposure to anti-coagulation, vs. those never started on anti-coagulation, and vs. those with continuous anti-coagulation, respectively. The secondary objective was to assess the effect of inequalities in anti-coagulant prescribing by comparing the risk of SSE and bleeding in those that received continuous OAC therapy with those that discontinued anti-coagulation in the context of a major bleeding event.

## Methods

### Study design

This was a retrospective observational cohort study of adults hospitalized with an incident AF event in Scotland.

### Data sources and cohort

Methods are reported in accordance with the REporting of studies Conducted using Observational Routinely-collected Data (RECORD) guidelines (see Supplementary material online).^12^ Public Health Scotland (PHS) provided access to fully anonymized data in support of a broader study which utilized routinely collected healthcare data to assess the comparative effectiveness of anti-coagulation for stroke prevention in people diagnosed with AF.

**Figure 1:**
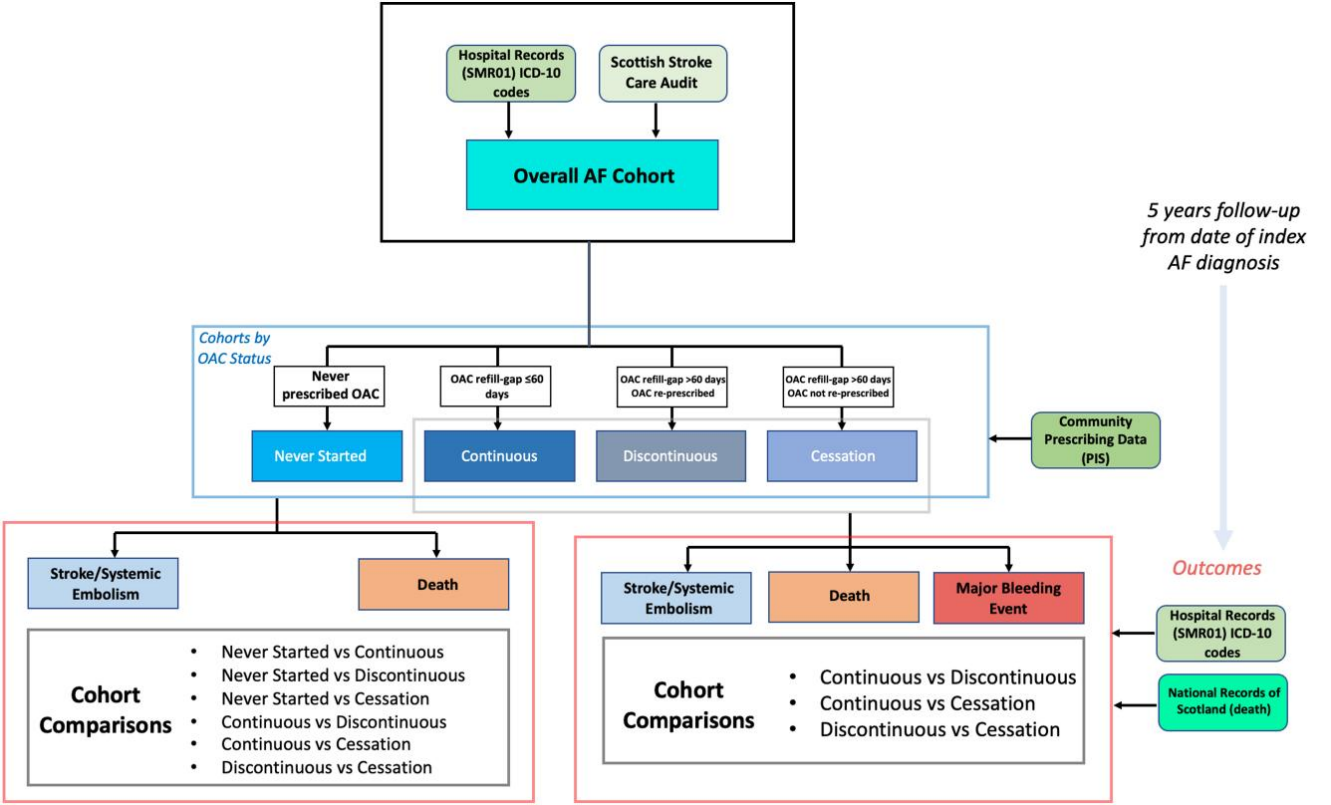
Outline of identification of atrial fibrillation population and formation of cohorts utilized in analyses, including sub-group analyses.

All adults aged 18 or older with a first diagnosis of AF between 1 January 2010 and 30 April 30 2016 with a CHA_2_DS_2_-VASC score (inclusive of 1 point for female sex) of 2 or greater were identified from the Scottish Morbidity Records (SMR) 01, which records inpatient and day case discharges for all specialities excluding psychiatry and obstetrics. The AF cohort in SMR01 was delineated by International Classification of Diseases (ICD)-10 coding in any diagnostic position (I48). We triangulated this with data from the Scottish Stroke Care Audit (SSCA), which collected information from all Scottish hospitals managing strokes, including whether AF had been diagnosed. There was considerable overlap when merging the data sets, with 16.9% of the records from SSCA unmatched with SMR01. Patient-level data linkage was undertaken with the National Records of Scotland (NRS) (mortality data) and Prescribing Information System (PIS) (prescribing data) (*Figure 1*). The NRS data set is the gold standard mortality data set in Scotland; whilst it is not validated, data entry is double-coded and previous audits indicate high data quality.^13^ Data linkage was also completed with SMR00 (outpatient appointments) to ensure relevant comorbidities were captured to inform calculation of CHA_2_DS_2_-VASC and HAS-BLED scores.

Stroke and bleeding events were measured using ICD-10 and Office of Population Censuses and Surveys (OPCS)-4 codes (see Supplementary material online, *Table S2*) in SMR01.^14^ SMR01 includes a Continuous Inpatient Stay (CIS) marker used to link episodes of care from distinct specialties or units within an unbroken period of admission to secondary care, which was used to identify the earliest date for clinical event and to avoid multiple counting of the same events.^15^ Inpatient data were available from 1 January 2005; the start date of the study was selected based on data availability allowing for a 5-year look-back period to identify those whose primary AF diagnosis preceded 1 January 2010. Those identified as having been diagnosed during the look-back period were excluded to avoid double-counting of the incident event. Data were available until 31 May 2021, and individuals were followed up until the event of interest and were censored at the end of the follow-up period, 5 years after the index date of AF diagnosis; follow-up was restricted to 5 years for all patients given that the likelihood of discontinuity in anti-coagulation would be greater in those with longer available follow-up compared with those that only had a minimum of 5 years of follow-up data available.

Our AF population was divided into four cohorts according to exposure to OAC: ‘never started’, ‘continuous OAC therapy’, ‘discontinuous OAC therapy’, and ‘cessation of OAC therapy’ (*Table 1*). Anti-coagulation is here defined as having been prescribed either warfarin or a direct oral anti-coagulant (DOAC) (apixaban, rivaroxaban, dabigatran, or edoxaban) for the first time following an AF event. Whilst DOAC prescriptions with dosages less than a minimum therapeutic threshold for prevention of thromboembolic events defined by the British National Formulary (BNF) were excluded, in the absence of availability of international normalized ratio (INR) data, it was assumed that doses of warfarin prescribed were sufficient for thromboprophylaxis. A look-back period of 30 days to the respective index AF diagnosis was implemented to ensure only individuals who were treatment naïve prior to commencing anti-coagulation were included in the analysis. Discontinuation of anti-coagulation was established according to the refill-gap method; those with a temporal gap between consecutive prescriptions of >60 days, not filled by the penultimate prescription, were defined as having discontinued.^16^ Prescriptions of OACs were determined from the PIS; this data set comprises records for the prescribing, dispensing, and reimbursement for all prescriptions dispensed by community pharmacies in Scotland.^17^ Prescriptions in Scotland are free of charge, with reimbursement data generated from pharmacies based on electronic or paper prescriptions after dispensing. Long-term repeat prescriptions, such as anti-coagulation, are typically prescribed at intervals of 28 or 56 days. To ensure that the indication for the anti-coagulant prescription was exclusively AF, individuals with valvular heart disease, a mechanical cardiac valve, or venous thromboembolism were excluded from analyses (see Supplementary material online, *Table S3*).

**Table 1:** Cohort definitions

Never started	Individuals with an incident AF event during the study that were not prescribed an oral anti-coagulant.
Continuous OAC therapy	Individuals with an incident AF event during the study that were prescribed an oral anti-coagulant with no refill gaps exceeding the defined threshold for discontinuation.
Discontinuous OAC therapy	Individuals with an incident AF event during the study that were prescribed an oral anti-coagulant, with a refill gap exceeding the defined threshold for discontinuation.
Cessation of OAC therapy	Individuals with an incident AF event during the study that were prescribed an oral anti-coagulant, with a refill gap exceeding the defined threshold for discontinuation, that received no further prescriptions of an oral anti-coagulant during the observation period.

Sub-group analyses evaluating risk of SSE, bleeding, and mortality were undertaken for people commenced on anti-coagulation that experienced a major bleeding event.

Data cleaning and pre-processing was undertaken according to established methodologies for routine healthcare data.^18^ Since <5% of records had missing data, and these appeared to be missing completely at random, imputation of the missing values was not completed; instead, a complete case analysis was undertaken, with those records excluded. Variables with missing data are detailed in Supplementary material online, *Table S4*.

The propensity score (PS)-based inverse probability of treatment weighting (IPTW) method was utilized to address potential confounding by indication, arising due to the lack of randomization.^19^ Inverse probability of treatment weighting was utilized rather than PS matching to avoid the exclusion of eligible subjects. Propensity score estimation was undertaken for the respective AF sub-groups to estimate the probability of anti-coagulant exposure status conditional upon the observed baseline characteristics of the AF population.^20^ Up to five PS models (never started vs. discontinuous OAC therapy, never started vs. cessation of OAC therapy, discontinuous OAC therapy vs. cessation of OAC therapy, continuous OAC therapy vs. discontinuous OAC therapy, continuous OAC therapy vs. cessation of OAC therapy) were thus generated for each outcome of interest. Logit models were used to estimate PSs and incorporated baseline characteristics which were either of prognostic relevance to the clinical outcomes or potentially predictive of anti-coagulation status. The models accounted for age (years), sex, and geographical location using an 8-fold urban–rurality classification, which was divided into three strata: urban (1–2), small and large towns (3–6), and rural (7–8). An indicator of socio-economic status, the Scottish Index of Multiple Deprivation (SIMD), was also included; this is a ranked scale of multiple deprivation for geographical locations and was divided into quintiles, such that one represents the most deprived and five denotes the least deprived areas, respectively. Propensity score also accounted for CHA_2_DS_2_-VASc at the time of the index AF event. Furthermore, PS for the ischaemic SSE outcomes also accounted for the following confounding variables: prior stroke/transient ischaemic attack (TIA), comorbidity (measured using the Charlson comorbidity index), and an electronic frailty score.^21^ Propensity score for the outcomes for major bleeding also accounted for anti-platelet prescriptions in the 2 years preceding the index AF event and time (days).

To implement the IPTW methodology, a weight indicative of the probability of either no exposure to anti-coagulation or continuous OAC therapy, and identical to the reciprocal of the aforementioned PS, was applied to individuals in those respective cohorts.^20^ Similarly, a weight equivalent to the reciprocal of one minus the PS was allocated to the discontinuous OAC therapy cohort. The risks of two significant clinical events associated with AF, stroke (both ischaemic and haemorrhagic) and major bleeding between discontinuation of anti-coagulation in individuals diagnosed with AF, was compared for those never prescribed anti-coagulation and for those with continuous exposure, using Cox proportional hazards regression. Doubly robust estimation was implemented by inclusion of the aforementioned PSs within the respective Cox proportional hazards regression models. To mitigate bias due to residual differences in the baseline covariates and redress possible covariate imbalance, the variables included to estimate PSs were incorporated into the adjusted models. Competing risk regression, using Fine and Gray proportional sub-hazards models, was implemented to establish the first clinical event (SSE or death and major bleed or death). Outcomes were evaluated using an intention-to-treat analysis.

Statistical analyses were completed using STATA version 16. Descriptive statistics for categorical variables are presented as the frequency and percentage in which they occurred and as mean and standard deviation for continuous variables. Regression analyses are presented as hazard ratios with 95% confidence intervals (CIs).

### Ethics

Ethical approval is not required for the analysis of secondary Scottish administrative healthcare data. However, the proposal for this study was reviewed and approved by the University of Glasgow Medicine, Veterinary Medicine and Life Sciences (MVLS) College Research Ethics Committee. This study was conducted in accordance with the International Society for Pharmacoepidemiology (ISPE) Guidelines for Good Pharmacoepidemiology Practices (GPP) and applicable regulatory requirements.

## Results

### Characteristics of the atrial fibrillation cohort

Of an overall cohort of 47 427 people with an incident diagnosis of AF between 1 January 2010 and 30 April 2016 and a CHA_2_DS_2_-VASC score of ≥2, those never prescribed anti-coagulations tended to be older, comprising a considerably greater proportion of people aged older than 85 (*Table 2*). Sex, urban–rurality score, CHA_2_DS_2_-VASc scores, and HAS-BLED scores were comparable between the cohorts. The cohort that never initiated anti-coagulant therapy had the greatest proportion of individuals in the most deprived SIMD quintile, whilst the continuous OAC therapy population had the greatest proportion of people categorized within the most affluent quintile. Furthermore, those aged ≥75, or with an elevated frailty score, were less likely to initiate anti-coagulation and, if prescribed, were more likely to discontinue.

**Table 2:** Descriptive statistics by anti-coagulation status

		Never started (*n* = 26 277)	Continuous (*n* = 7934)	Discontinuous (*n* = 9107)	Cessation (*n* = 4109)
**Age**	≤55	429	188	208	65
	%	1.63	2.37	2.28	1.58
	56–75	6311	3620	4216	1213
	%	24.02	45.63	46.29	29.52
	76–85	10 634	3242	3862	1960
	%	40.47	40.86	42.41	47.70
	>85	8903	884	821	871
	%	33.88	11.14	9.02	21.20
	Mean (SD)	80.72 (9.52)	75.36 (8.6)	74.98 (8.39)	78.59 (8.58)
**Sex**	Female	15 182	4496	5141	2202
	%	57.78	56.67	56.45	53.59
	Male	11 095	3438	3966	1907
	%	42.22	43.33	43.55	46.41
**CHA_2_DS_2_-VASC score**	2	6404	2292	2970	994
	%	24.37	28.89	32.61	24.19
	3	9041	2378	2848	1286
	%	34.41	29.97	31.27	31.30
	4	6066	1684	1822	924
	%	23.08	21.23	20.01	22.49
	5	3135	1035	985	601
	%	11.93	13.05	10.82	14.63
	6	1255	428	373	224
	%	4.78	5.39	4.10	5.45
	7	322	107	100	68
	%	1.23	1.35	1.10	1.65
	8	47	10	9	12
	%	0.18	0.13	0.10	0.29
	9	7	0	0	0
	%	0.03	0.00	0.00	0.00
**HAS-BLED score**	0	121	53	69	20
	%	0.46	0.67	0.76	0.49
	1	4698	1628	1757	789
	%	17.88	20.52	19.29	19.2
	2	10 237	3091	3735	1529
	%	38.96	38.96	41.01	37.21
	3	7658	2299	2594	1222
	%	29.14	28.98	28.48	29.74
	4	2899	744	827	476
	%	11.03	9.38	9.08	11.58
	≥5	664	119	125	73
	%	2.53	1.5	1.37	1.78
**Grouped Charlson index**	0	10 031	4223	4797	1870
	%	38.17	53.23	52.67	45.51
	1	5407	1660	1985	865
	%	20.58	20.92	21.80	21.05
	2	10 839	2051	2325	1374
	%	41.25	25.85	25.53	33.44
**Urban–rurality**	1–2 (city)	18 620	5374	6204	2797
	%	70.86	67.73	68.12	68.07
	3–6 (towns)	3337	1057	1205	550
	%	12.70	13.32	13.23	13.39
	7–8 (rural)	4320	1503	1698	762
	%	16.44	18.94	18.64	18.54
**SIMD**	1	6153	1688	1933	857
	%	23.42	21.28	21.23	20.86
	2	6328	1796	2038	996
	%	24.08	22.64	22.38	24.24
	3	5349	1642	1946	911
	%	20.36	20.70	21.37	22.17
	4	4552	1476	1689	708
	%	17.32	18.60	18.55	17.23
	5	3895	1332	1501	637
	%	14.82	16.79	16.48	15.50
**Prior stroke**	%	1615	367	346	171
		6.15	4.63	3.80	4.16
**Previous anti-platelet**	%	15 523	4146	5134	2318
		59.07	52.26	56.37	56.41
**Frailty score**	Low risk <5	7114	4204	4174	1045
		27.07	52.99	45.83	25.43
	Moderate risk 5–15	10 081	2679	3172	1543
		38.36	33.77	34.83	37.55
	High risk >15	9082	1051	1761	1521
		34.56	13.25	19.34	37.02

The rate of discontinuation appears to be greatest at the extremes of the CHA_2_DS_2_-VASC score, with rates of 63.4% and 63.5% for scores of 2 and 3 and 63.6% and 67.7% for scores of 7 and 8, respectively. Initiation and continuity of anti-coagulation improved according to year of AF diagnosis (see Supplementary material online, *Figure S1*). Direct oral anti-coagulants replaced warfarin as the predominant OAC prescribed over the duration of the study (see Supplementary material online, *Figure S2*) although OAC discontinuity was comparable between OAC types (see Supplementary material online, *Figure S3*).

### Stroke/systemic embolism risk

The percentage diagnosed with stroke or systemic embolism during the 5 years after AF diagnosis was highest in the discontinuous OAC therapy cohort (16.54%) and lowest in those that received continuous anti-coagulation (5.99%).

**Figure 2:**
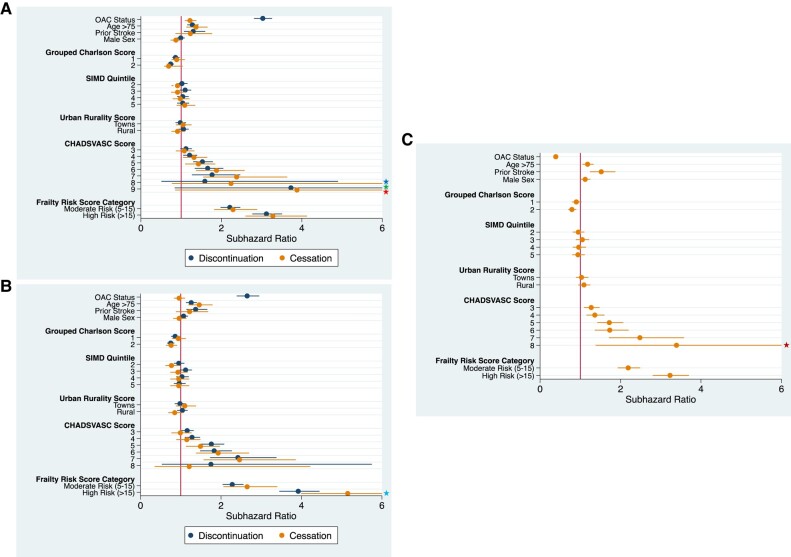
Stroke/systemic embolism risk. (*A*) Never started vs. discontinuous and cessation. (*B*) Continuous vs. discontinuous and cessation. (*C*) Discontinuous vs. cessation reference. Truncated upper confidence intervals: 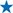
16.49 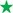
6.48 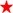
17.75 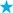
6.66 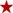
8.43.

People receiving discontinuous OAC therapy had an increased risk of stroke during the 5-year follow-up period from an initial AF diagnosis, compared with those that never started OAC [subhazard ratio (SHR) 3.03; 2.81–3.26] (*Figure 2A*) and those with continuous anti-coagulant prescriptions (SHR 2.65; 2.39–2.94) (*Figure 2B*). Cessation was associated with greater stoke risk than individuals that never started anti-coagulation (SHR 1.22; 1.22–1.38) (*Figure 2B*) but lower stroke risk than people in the discontinuous OAC therapy cohort (SHR 0.39; 0.34–0.44) (*Figure 2C*). There was no significant difference in stroke risk between people that permanently discontinued anti-coagulation and those with continuous OAC therapy. Prior stroke, age >75, comorbidities, and elevated frailty risk score were significantly associated with a higher risk of stroke. Compared with a reference score of 2, all CHA_2_DS_2_-VASC scores were associated with a greater risk of SSE. Compared with the most deprived SIMD quintile, the second and third quintiles had similar stroke risk, whilst the fourth and fifth quintiles were associated with a reduced risk of stroke, which was statistically significant. Mortality risk in this population is reported in Supplementary material online, *Figure S4*.

**Figure 3:**
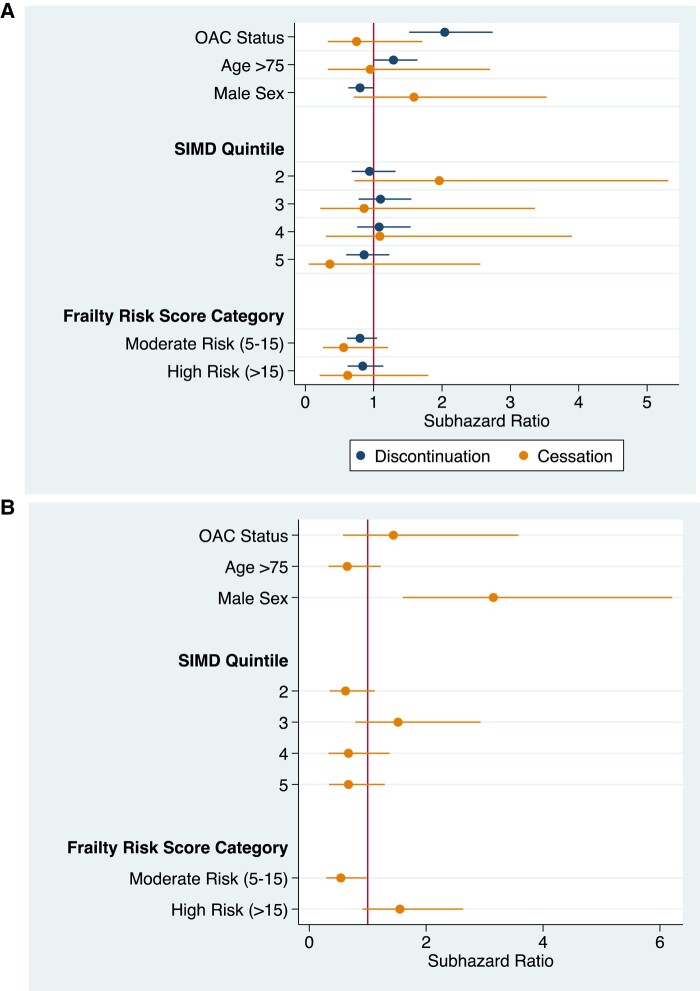
Stroke/systemic embolism risk following major bleeding event. (*A*) Continuous vs. cessation and discontinuous. (*B*) Discontinuous vs. cessation reference. Analyses were also adjusted for time off anti-coagulation.

Discontinuation of OAC therapy was associated with an increased risk of stroke in people that had a prior bleeding event compared with those with continuous OAC therapy (SHR 2.04; 0.52–2.74) (*Figure 3A*). Sex, age older than 75, SIMD quintile, urban–rurality classification, time off anti-coagulation, and frailty score were not significant mediators of stroke risk in this cohort. Cessation was not associated with a statistically significant difference in risk of stroke compared with those with discontinuous or continuous OAC therapy (*Figure 3A* and *B*).

### Major bleed analyses

#### Anti-coagulation prescriptions

Warfarin was the most common anti-coagulant in our cohort, prescribed to 71.6% of people. Apixaban was the mostly commonly prescribed DOAC (15%), followed by rivaroxaban (11.5%); dabigatran (1.3%) and edoxaban (0.53%) were utilized more rarely.

#### Stroke/systemic embolism risk

During 5 years of follow-up from index AF diagnosis, the highest percentage of stroke and systemic embolism events in individuals with a prior major bleed event occurred in the discontinuous OAC therapy (33.63%) and cessation (33.33%) cohorts; the continuous OAC therapy population had lowest proportion of stroke events (14.76%).

#### Bleeding risk

Recurrent bleeding was most frequent in individuals that discontinued anti-coagulation, either temporarily (27.68%) or permanently (20.5%), or were never prescribed it (22.98%).

**Figure 4:**
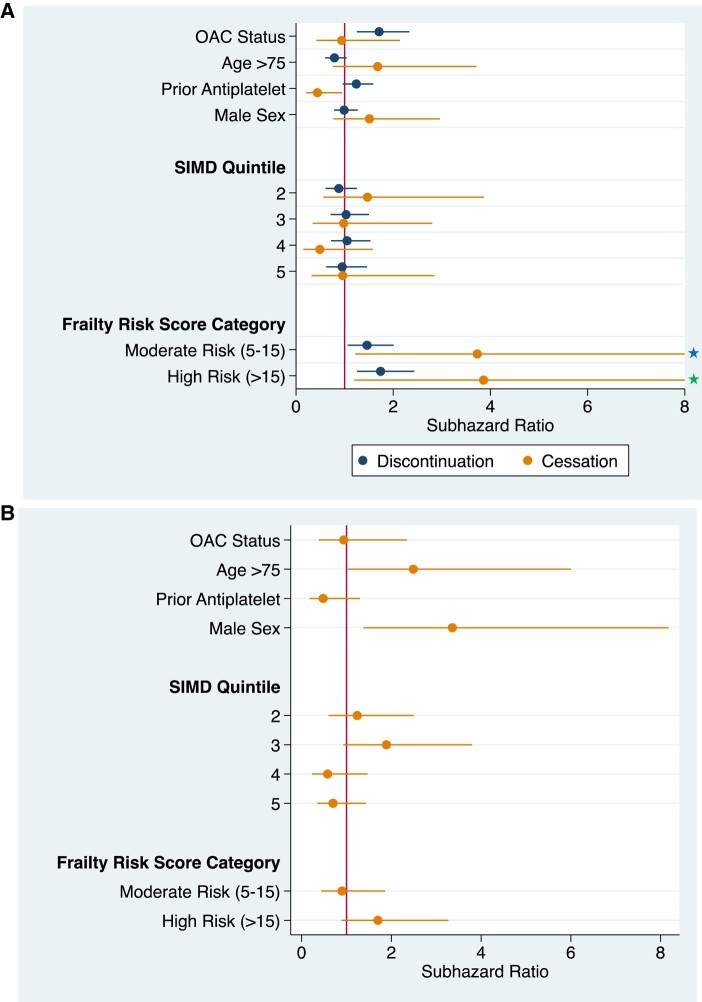
Recurrent bleeding risk. (*A*) Continuous vs. cessation and discontinuous. (*B*) Discontinuous vs. cessation reference. Truncated upper confidence intervals: 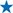
11.41 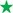
12.49. Analyses were also adjusted for time off anti-coagulation.

The discontinuous OAC therapy cohort had a greater risk of a bleeding event compared with those that persisted with anti-coagulation (SHR 1.71; 95% CI: 1.25–2.33) (*Figure 4A*). The highest category of frailty risk was associated with an increased risk of recurrent bleeding. Mortality risk in this population is reported in Supplementary material online, *Figure S5*.

## Discussion

This retrospective cohort study utilized national linked individual patient data to compare the risks of SSE and bleeding in AF according to anti-coagulant exposure, from which there are multiple key observations. Firstly, despite the high thromboembolic risk in our population, the rate of anti-coagulation was low, and the rate of discontinuation was high, although this improved over the duration of our study. There were considerable inequalities in OAC prescribing; lower socio-economic, elevated frailty score, and age >75 were associated with anti-coagulation not being commenced and non-continuous OAC prescribing. Furthermore, the SSE risk associated with discontinuation of anti-coagulation appears to be at least equivalent to, if not greater than, never commencing anti-coagulation. Finally, our analyses also indicate that discontinuation or cessation of OACs is not protective with regard to the risk of recurrent bleeding.

### Oral anti-coagulant prescribing

Although our population had an elevated SSE risk, a considerable proportion, 55.4%, never commenced warfarin or a DOAC, which is consistent with prior studies highlighting sub-optimal anti-coagulation amongst the AF population. Indeed, a recent study by Lee *et al.*^22^ reported that 49.7% of those diagnosed with AF admitted to the hospital in Scotland between 2010 and 2019 were not anti-coagulated, although this had improved from 63.8% in 2010 to 35.5% in 2019. They found that women were less likely to be anti-coagulated, although this disparity was mitigated by the increasing prevalence of anti-coagulation with DOACs. Although our data did not include information on the rationale for decision-making on anti-coagulation, De Breucker *et al.*^23^ previously reported that the absence of anti-coagulation in older adults is likely due to functional or cognitive impairment, fall risks, malnutrition, or depression.

Whilst sustained endeavours to ensure that anti-coagulation is appropriately initiated at diagnosis are clearly integral, it is also concerning that despite AF being a lifelong condition, requiring ongoing thromboprophylaxis, only 37.51% of our population received continuous OAC therapy. We observed a discontinuation rate of 62.49% with comparable discontinuation between DOACs and warfarin, which is consistent with prior studies. Indeed, a retrospective study by Baker *et al.*^24^ of discontinuation (defined as a temporal gap in prescriptions of >30 days) in 41 864 individuals with AF in the USA prescribed DOACs reported discontinuation rates of 60.3%, 52.8%, and 62.9% for rivaroxaban, apixaban, and dabigatran, respectively. Furthermore, in a retrospective cohort study in the USA of 12 129 adults with AF, 47% discontinued OAC, which occurred within an average of 120 days; the first discontinuation often occurs early after the initial prescription.^25^

Some studies considered adherence and persistence, rather than, or in addition to, discontinuation explicitly, in their analyses. Indeed, Dhamane *et al.*^26^ conducted a retrospective analysis of non-persistence, defined as treatment switching, or a refill gap of 60 days or greater, in over one million people with AF prescribed anti-coagulants in the USA. At 1 year, the cumulative incidence of non-persistence was 51.3%, 58.9%, 51.3%, and 52.2%, for warfarin, apixaban, rivaroxaban, and dabigatran, respectively. Few studies have evaluated the cessation of anti-coagulation; a prospective cohort study in Italy assessing cessation of DOACs in 1305 adults with AF reported that 15.4% were no longer anti-coagulated after 1 year of follow-up; >60% discontinued within the initial 6 months.^27^

Our analyses also suggest considerable inequalities in OAC prescribing, which are essential to address, given that non-initiation and discontinuity of anti-coagulation are associated with poorer clinical outcomes, including a greater SSE risk. For example, those living in the most deprived areas were most likely to have never commenced anti-coagulation, whilst those living in the most affluent areas were most likely to have been prescribed anti-coagulation. A previous study of stroke survivors with AF in Scotland found that the most deprived SIMD quintiles were associated with an absence of anti-coagulation.^28^ Furthermore, those in the least deprived quintile were most likely to have continuous anti-coagulation, which is consistent with international studies.^29,30^ However, in a recent study of trends in OAC prescribing in England, a higher socio-economic status appeared to be associated with non-adherence.^31^

### Stroke risk following oral anti-coagulant discontinuation

Our analyses indicate that OAC discontinuation is associated with a significantly increased SSE risk in adults with AF, compared with those that are continuously anti-coagulated. Indeed, Toorop *et al.*^32^ evaluated OAC persistence in a Dutch AF cohort, defined as a gap of <100 days between the final OAC prescription and the study end date, and found that non-persistence was associated with a 58% increase in the risk of ischaemic stroke compared with those with continuous OAC exposure. Similarly, Rodriguez *et al.*^33^ conducted a nested case-control analysis of electronic health records in the UK and Denmark, matching incident cases of ischaemic stroke to controls by age and sex, and found that those that discontinue OAC have a risk of stroke two to three times greater than those with continuous treatment.

In our population, temporary discontinuation of anti-coagulation was also associated with a greater thromboembolic risk than in people that never initiated OAC therapy and in those that permanently discontinued. Discontinuation is ostensibly associated with a period of significant clinical risk during which individuals require active, dynamic monitoring. Indeed, rebound hypercoagulability has been postulated to occur transiently in the aftermath of discontinuation, associated with raised blood markers of thrombin production.^34^ A cohort study in South Korea reported that discontinuation of DOACs was associated with greater stroke severity at initial presentation, per the National Institute of Health Stroke Scale (NIHSS), than those that discontinued warfarin and those never commenced on anti-coagulation which they postulated was due to this hypercoagulable state.^35^

### Outcomes following oral anti-coagulant discontinuation in the context of a major bleeding event

This study found that, in the context of a major bleeding event, individuals that discontinued anti-coagulation had an increased risk of SSE and of a further bleeding event compared with those continuously anti-coagulated. Furthermore, our analyses indicated no significant difference in the risk of a subsequent bleed between those that permanently discontinued compared with those that continued anti-coagulation, in those who had experienced a major bleeding event. Whilst the respective cohorts reflect anti-coagulant prescribing status at the time of the relevant clinical events, there is considerable heterogeneity within the discontinuation cohorts, with regard to the number of, and duration of, discontinuations before and after the event. Thus, the analysis and interpretation of this sequencing is challenging, particularly in the absence of information on the rationales for anti-coagulant prescribing decisions within our data set. Ewen *et al.*^36^ previously found no difference in rates of bleeding events between people with continuous and discontinuous anti-coagulation, although this was in a primary care setting with a shorter follow-up duration of 12 months. Several prior cohort studies, and a meta-analysis, have reported that recommencing anti-coagulation following a bleed is associated with a lower risk of ischaemic stroke and all-cause mortality to those for whom anti-coagulation remained withheld, with no statistically significant difference in the risk of bleeding.^5,37,38^ However, these have typically focused on a single type of bleeding event, for example, gastrointestinal bleeding, or only considered these outcomes in the short term. Consensus on the optimum time to reinitiate anti-coagulation following a major bleeding event is currently lacking; the American Heart Association recommends re-initiation of anti-coagulation within 7–10 days following an intracranial haemorrhage, whilst others have proposed 10 weeks.^39^ Acutely, the risk of further bleeding is likely greater than the risk of stroke or systemic embolism, whilst in the longer term, the risk of thromboembolism may exceed that of a recurrent bleed.

### Strengths and limitations

A key strength of our study is the use of national linked data to understand, over a relatively long follow-up period, patterns and inequalities in anti-coagulant prescribing in a real-world AF population and the effects of discontinuation of anti-coagulation on clinical outcomes. Our results are generalizable to similar AF populations, particularly in those for which there is universal healthcare provision.

However, our study has several possible limitations. Firstly, given the retrospective study design, there may have been unmeasured confounding which could have biased our effect estimates, which could not feasibly be investigated within the context of an RCT.^40^ However, in the absence of randomized controlled trials, which would be unethical and infeasible, retrospective observational analyses are the optimal alternative to generate evidence in this context.

Second, since SMR01 and SSCA only include diagnoses relevant to hospital admissions, AF diagnoses in primary care were not captured in our data.^41^ Furthermore, a survey of English general practices reported that half of people with AF were managed exclusively in primary care; comparatively, our secondary care AF population is likely at greater risk of the outcomes measured.^42^

Clinical miscoding is a possible risk of utilizing data derived from administrative healthcare records. Furthermore, PIS only captures primary care prescribing data, such that secondary care prescriptions of anti-coagulants may thus create a temporal gap within the PIS data. A refill gap of 60 days was thus selected to afford sufficient time for community prescribing to have resumed following a secondary care admission, to reduce the potential for erroneous assignment of people to the discontinuous cohort following hospital discharge, since the reason for discontinuation is not recorded in PIS. Assuming anti-coagulation continued to be prescribed and dispensed, people with poor adherence, and potentially those with sub-therapeutic doses of warfarin, would be classified within the continuous cohort. Our data set also does not contain information related to prescriber decision-making or on procedures such as AF ablation or cardioversion, and thus, the rationales for OAC non-prescribing, initiation, and discontinuation are uncertain.

A further limitation of the study is that the study was undertaken during a transition period in anti-coagulant prescribing, in which DOACs replaced warfarin as the preferred anti-coagulant in people with AF. Indeed, DOACs have an improved safety profile and do not have the monitoring requirements of warfarin and thus may be associated with greater adherence.

Finally, given the heterogeneous patterns of adherence for anti-coagulation, our definition for discontinuation may not have adequately captured the risks of our outcome measures for all individuals in this cohort. Indeed, individuals within this cohort may have had significantly different time periods in which they were not anti-coagulated.

### Future research

Harmonization of definitions and methodologies in the evaluation of discontinuation of anti-coagulation would be highly valuable in aiding comparisons since there is significant heterogeneity in the existing literature.

Given the under-utilization of thromboprophylaxis, and high discontinuation rates observed in our analyses and in the wider literature, further research is critical to elucidate the rationales underlying decision-making more clearly, by clinicians and patients, around anti-coagulant prescribing. Indeed, this will allow the development of targeted interventions aimed at promoting appropriate OAC initiation on diagnosis of AF and mitigating OAC discontinuation, such that stroke prevention may be optimized; patient-centred approaches which promote shared decision-making between clinicians and patients are valuable in supporting adherence.^43^ This is particularly important since there is increasing evidence that the lack of monitoring for DOACs, whilst convenient, may be deleterious to persistence; monitoring appointments afford healthcare professionals the opportunity for continued patient education to reinforce the rationale for anti-coagulation.^44^

Further research is necessary to establish an optimal approach to the management of anti-coagulants in adults with AF in the context of major bleeding events, given the lack of consensus amongst clinicians and consequent variation in real-world clinical practice. This would support the identification of individuals who may safely recommence anti-coagulation at the appropriate timepoint following a bleed, as well as those in whom future anti-coagulation is potentially contra-indicated, optimizing clinical outcomes.

## Conclusions

Considerable inequalities in OAC prescribing exist for people with AF in Scotland, which is of particular significance since non-initiation and discontinuity of anti-coagulation are associated with poorer clinical outcomes, including increased SSE risk.

## Supplementary Material

oeae016_Supplementary_Data

## Data Availability

The data are confidential, and analyses were subject to disclosure control by PHS. Access to the data may be requested by application to PHS via the Public Benefit and Privacy Panel.

## Lead author biography

Ryan joined the Health Economics and Health Technology Assessment (HEHTA) team at the University of Glasgow in 2022. He obtained his medical degree from the University of Edinburgh in 2018 and completed an MSc in Precision Medicine and Pharmacological Innovation at the University of Glasgow in 2020. Ryan’s current work is focused on the use of real-world evidence in health technology assessment (HTA), particularly within oncology and cardiovascular disease, and evidence synthesis. His research interests also include analysis of linked health data, the economics of precision medicine, and economic evaluation alongside clinical trials, particularly within the field of oncology.
